# Lithium in the prevention of suicide in adults: systematic review and meta-analysis of clinical trials

**DOI:** 10.1192/bjo.2022.605

**Published:** 2022-11-17

**Authors:** Natalie B. Riblet, Brian Shiner, Yinong Young-Xu, Bradley V. Watts

**Affiliations:** Veterans Affairs Medical Center, White River Junction, Vermont, USA; and Geisel School of Medicine at Dartmouth College, Hanover, New Hampshire, USA; Veterans Affairs Medical Center, White River Junction, Vermont, USA; Geisel School of Medicine at Dartmouth College, Hanover, New Hampshire, USA; and National Center for PTSD, White River Junction, Vermont, USA; Veterans Affairs Medical Center, White River Junction, Vermont, USA; Geisel School of Medicine at Dartmouth College, Hanover, New Hampshire, USA; and Veterans Rural Health Resource Center, Veterans Affairs Medical Center, White River Junction, Vermont, USA

**Keywords:** Lithium, suicide prevention, anti-manic agents, randomised controlled trials, systematic review

## Abstract

Controversy exists regarding the efficacy of lithium for suicide prevention. Except for a recent trial that enrolled over 500 patients, available trials of lithium for suicide prevention have involved small samples. It is challenging to measure suicide in a single randomised controlled trial (RCT). Adding a single large study to existing meta-analyses may provide insights into lithium's anti-suicidal effects. We performed a meta-analysis of RCTs comparing lithium with a control condition for suicide prevention. MEDLINE and other databases were searched up to 30 November 2021. Efficacy was assessed by calculating the summary Peto odds ratio (OR) and incidence rate ratio (IRR) with 95% confidence intervals. Among seven RCTs, the odds of suicide were lower among patients receiving lithium versus control (OR = 0.30, 95% CI 0.09–1.02; IRR = 0.22, 95% CI 0.05–1.05), although the findings were still not statistically significant. The role of lithium in suicide prevention remains uncertain.

Suicide remains a global public health problem.^1^ Clinicians and patients are in critical need of effective interventions to prevent suicide but researchers have found few interventions with proven efficacy.^2,3^ In addition, it is difficult to study suicide in a clinical trial.^4^ Generally, studies require well over 1000 patients to detect an effect.^2^ To bolster statistical power and ability to detect a true effect, researchers increasingly use approaches such as meta-analysis.^2^

Although lithium has been viewed as a promising strategy to prevent suicidal behaviour since the 1970s,^5^ there remains controversy about whether it can prevent suicide.^2,5,6^ In a meta-analysis of four randomised controlled trials (RCTs) of lithium versus placebo, Cipriani et al (2013) found that lithium was more protective against suicide than placebo (*n* = 485; odds ratio OR = 0.13, 95% CI 0.03–0.66).^6^ In a subsequent meta-analysis of six RCTs of lithium versus control (placebo, usual care or waiting list), Riblet et al (2017) found that the odds of suicide were lower with lithium, but the results were not significant (*n* = 619; OR = 0.23, 95% CI 0.05–1.02; incidence rate ratio IRR = 0.14, 95% CI 0.00–9.41).^2^

A key limitation of existing meta-analyses of lithium for suicide prevention is the reliance on relatively small population samples.^2,6^ This has yielded wide confidence intervals^2^ and limited the interpretation of positive^6^ and negative^2^ findings. Because few clinical interventions have proven efficacy in preventing suicide^2,3^ and the anti-suicidal effect of lithium is debatable,^2,5,6^ it is crucial that researchers report on evidence as it emerges in the field. This knowledge can inform clinical practice and future research.

Given the recent publication of the largest ever RCT of lithium for suicide prevention,^7^ we performed a new meta-analysis of lithium use in the prevention of suicide in adults. A review of current evidence will provide healthcare providers, policymakers and researchers with an improved understanding of the clinical use (and future direction of research) of lithium for suicide prevention.

## Method

As described in the supplementary Methods (available at https://doi.org/10.1192/bjo.2022.605) we developed a study protocol to identify studies, abstract study data, assess study quality and determine the effect of lithium on suicide. The protocol was posted on PROSPERO (CRD42022295822).

We included studies that randomly assigned adult patients to lithium or a control condition (usual care, placebo or waiting list). We searched MEDLINE (via Ovid), Embase, CINAHL, the Cochrane Library's CENTRAL, and PsycINFO from 1 January 2015 to 30 November 2021 to identify published (including ‘Epub ahead of print’) articles that met our inclusion criteria.

We evaluated the relationship between lithium and suicide using the Peto method. We calculated summary OR with 95% CI and *P*-values. We used a Poisson regression model with random effects to calculate an IRR for suicides over person-years. We applied GRADE methodology to determine the effect of the quality of the evidence on our findings. All analyses were conducted using STATA version 17 for Windows (StataCorp). Because this was a study of published literature, ethics approval and informed consent were not required.

## Results

As shown in supplementary Fig. 1, we identified seven RCTs that randomly assigned adult patients to lithium or a control condition (placebo or usual care) and reported on suicide. The seven studies were all conducted in Europe and North America and enrolled patients with major depressive disorder or bipolar disorder (supplementary Table 1).

**Fig. 1:**
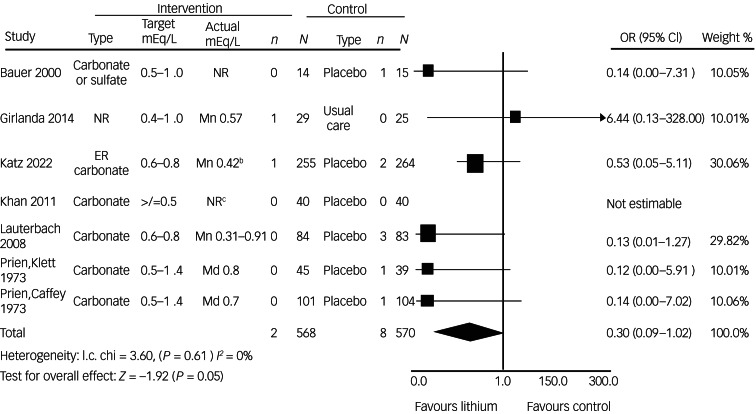
Forest plot of the odds of suicide among patients randomised to lithium versus a control condition. a. References for the seven studies appear in the supplementary Methods. b. Participants with bipolar affective disorder had a mean lithium concentration of 0.54 mEq/L at 3 months, whereas those with major depressive disorder had a mean concentration of 0.46 mEq/L at 3 months. c. 11 patients had therapeutic levels and 29 patients had non-therapeutic levels of lithium. ER, extended release; *n*, number of suicide events; *N*, number of participants; Md, median, Mn, mean; NR, not reported; χ^2^, Cochran's *Q*.

Within the identified studies, the odds of suicide were lower for the 568 patients allocated to lithium than for the 570 allocated to a control condition (OR = 0.30, 95% CI 0.09–1.02, *P* = 0.05) (Fig. 1). The IRR favoured lithium (IRR = 0.22, 95% CI 0.05–1.05, *P* = 0.06). The findings, however, were not significant.

We did not observe substantial or significant heterogeneity among the included studies (Cochran's *Q* = 3.60, *I*^2^ = 0%, *P* = 0.61). On visual inspection, we identified one study with an extremely wide confidence interval. This was the only study that included treatment as usual as a comparator and the results favoured the control condition.

In a risk of bias assessment, we identified some concerns about the potential effect of study assignment and adherence (supplementary Table 2). This was usually because the authors reported that some participants did not adhere to the study drug. Several studies reported high rates of attrition. A few studies encountered problems with recruitment.

A visual inspection of the funnel plot suggested no evidence of publication bias. The summary estimate included a smaller study that reported a negative result (Fig. 1; supplementary Fig. 2).

According to our GRADE analysis, the certainty of the evidence in favour of lithium was moderate. The finding was important as it pertains to mortality (supplementary Table 3).

## Discussion

Our meta-analysis showed that lithium was associated with a 70% lower odds of suicide. Although the finding is promising, the results were not significant. Our results corroborate those of prior meta-analyses^2^ as well as that of an individual large trial^7^ suggesting that there is insufficient evidence to support that lithium has an anti-suicidal effect. Yet the results of our review emphasise the need to study further the role of lithium for suicide prevention. First, unlike prior meta-analyses,^2^ by including over 1000 patients in our analysis, we were able to generate a substantially narrowed confidence interval around the IRR estimate. Second, we made a conservative decision to exclude from the analysis a single death due to opioid overdose.^7^ There was no indication in the study that this death was classified as a suicide.^7^ Experts in the field have raised concerns that some overdose deaths may be misclassified suicides.^8^ Related to these concerns, in the suicide prevention literature there is growing interest in addressing self-injury mortality (defined as suicide deaths by any method plus estimated deaths due to accidental or undetermined drug overdose) rather than suicide mortality alone.^8,9^ The inclusion of that overdose death^7^ in our study would have generated a significant finding in favour of lithium for self-injury mortality prevention (OR = 0.28, 95% CI 0.08–0.90; IRR = 0.20, 95% CI 0.04–0.93). Lastly, it is worth recalling that a *P*-value of 0.05 or 0.06 indicates a 5–6% possibility that our results were due to chance rather than an anti-suicide effect of lithium.

There are several proposed theories to explain why lithium might be effective at preventing suicide.^5^ One of the many possibilities includes lithium's role as a mood stabiliser.^5^ There is some evidence in the literature that links impulsivity to suicidal behaviour.^5,10^ For example, in a 14-year naturalistic study of patients with affective illness, Maser et al (2002) found that impulsivity was one of the best predictors of suicide after 1-year follow-up (sensitivity 74%, specificity 82%).^10^

It is a strength of our review that we have produced summary estimates for over 1100 patients and improved the precision of the estimate. The trials, however, varied in their target (and actual) lithium levels. Some studies reported poor recruitment, low treatment adherence or high study attrition. Most studies followed patients for less than 1 year. Finally, the generalisability of our results to populations outside of Europe and North America, or patients without depression or bipolar disorder, remains unclear.

Because the findings from our meta-analysis raise questions about the benefit of lithium for suicide prevention, we caution clinicians, researchers and policymakers against dismissing further examination of lithium for suicide prevention.

## Data Availability

The data sources used in this study are available from the corresponding author on reasonable request.

## Supplementary material

For supplementary material accompanying this paper visit http://doi.org/10.1192/bjo.2022.605. click here to view supplementary material

## References

[1] Zheng Y, Molassiotis A, Tyrovolas S, Yip PSF. Epidemiological changes, demographic drivers, and global years of life lost from suicide over the period 1990–2019. Suicide Life Threat Behav 2022; 52: 439–51. 35137457 10.1111/sltb.12836

[2] Riblet NBV, Shiner B, Young-Xu Y, Watts BV. Strategies to prevent death by suicide: meta-analysis of randomised controlled trials. Br J Psychiatry 2017; 210: 396–402. 28428338 10.1192/bjp.bp.116.187799

[3] Platt S, Niederkrotenthaler T. Suicide prevention programs: evidence base and best practice. Crisis 2020; 41(suppl 1): S99–124. 32208762 10.1027/0227-5910/a000671

[4] Perlis RH. Hard outcomes: clinical trials to reduce suicide. Am J Psychiatry 2011; 168: 1009–11. 21969041 10.1176/appi.ajp.2011.11081250

[5] Lewitzka U, Severus E, Bauer R, Ritter P, Müller-Oerlinghausen B, Bauer M. The suicide prevention effect of lithium: more than 20 years of evidence-a narrative review. Int J Bipolar Disord 2015; 3(1): 32. 26183461 10.1186/s40345-015-0032-2 PMC4504869

[6] Cipriani A, Hawton K, Stockton S, Geddes JR. Lithium in the prevention of suicide in mood disorders: updated systematic review and meta-analysis. BMJ 2013; 346: f3646. 23814104 10.1136/bmj.f3646

[7] Katz IR, Rogers MP, Lew R, Thwin SS, Doros G, Ahearn E, Lithium treatment in the prevention of repeat suicide-related outcomes in veterans with major depression or bipolar disorder: a randomized clinical trial. JAMA Psychiatry 2022; 79: 24–32. 34787653 10.1001/jamapsychiatry.2021.3170 PMC8600458

[8] Rockett IRH, Caine ED. Reconciling suicides with “accidental” drug-intoxication deaths: a behaviorally based definition of self-injury mortality. Am J Public Health 2020; 110: 73–4. 31800285 10.2105/AJPH.2019.305422 PMC6893324

[9] Rockett IR, Lilly CL, Jia H, Larkin GL, Miller TR, Nelson LS, Self-injury mortality in the United States in the early 21st century: a comparison with proximally ranked diseases. JAMA Psychiatry 2016; 73: 1072–81. 27556270 10.1001/jamapsychiatry.2016.1870 PMC5482223

[10] Maser JD, Akiskal HS, Schettler P, Scheftner W, Mueller T, Endicott J, Can temperament identify affectively ill patients who engage in lethal or near-lethal suicidal behavior? A 14-year prospective study. Suicide Life Threat Behav 2002; 32: 10–32. 11931008 10.1521/suli.32.1.10.22183

