# Supplementary material for: Lithium in the prevention of suicide in adults: systematic review and meta-analysis of clinical trials
Source: BJPsych Open. 2022 Nov 17;8(6):e199. doi: 10.1192/bjo.2022.605 (PMC9707499; doi:10.1192/bjo.2022.605)
Supplement: Supplementary file 1 [file bjosup.zip › S2056472422006056sup001.docx]

**Supplementary Table 1 [Desired position – horizontal]** Characteristics of included studies and ongoing trials.

| **Author/Year** | **Status** | **Country** | **Setting** | **Population** | **Excluded MH Conditions** | **Total N** | **Percent Female** | **Follow-up, m** | **Follow-up, PY**  **Li/Control** | **Mortality Outcomes (N-cause)** | **Percent Attrition** | **Industry Funding** |
| --- | --- | --- | --- | --- | --- | --- | --- | --- | --- | --- | --- | --- |
| ***Published Studies*** | | | | | | | | | | | | |
| Bauer’00 | Completed | Germany | Inpatient Outpatient | MDD, fail Rx* and stabilized on Lithium, no acute suicidal ideation | History of hypomanic or manic episode, Other Axis I Diagnosis | 29 | 58.6 | 4 | 4.6/5.0 | Li (no deaths); Placebo (1-suicide) | 0.0 | Yes** |
| Girlanda’14 | Trial did not achieve recruitment goals | Italy | Inpatient or Outpatient | Treatment resistant MDD + deliberate self-harm in previous 12 months | Any concurrent Axis I Diagnosis | 56 | 63.0^χ^ | 12 | 29/25 | Li (1-suicide); Placebo (no deaths) | 9.3 | No |
| Katz’22 | Terminated (futility) | USA | VA Inpatient Outpatient | BPAD or MDD plus suicidal behavior or admitted to prevent suicidal behavior within 6 months | Schizophrenia, unstable SUD; 6 or more SA | 519 | 15.8 | 13 | 218.7/ 231.5 | Li (1- suicide); Placebo (2-suicide, 1- opioid overdose) | 48.2 | No |
| Khan’11 | Completed | USA | Outpatient | MDD, dysthymia, or depression + suicidal behavior^§^ | None | 80 | 55.0 | 1.4 | 4.6/4.6 | No deaths | 0.0 | Yes |
| Lauterbach’08 | Terminated  (recruitment) | Germany | ER | Depression + suicide attempt within 3m | Schizophrenia, BPD with severe self-harm, SUD, clear indication for long term Li | 167 | 57.5 | 12 | 55.2/46.1 | Li (no deaths); Placebo (3-suicide) | 68.9 | Yes |
| Prien, Klett ’73 | Completed | USA | VA & non-VA  Inpatient | Depressed (MDD or BPAD) + stabilized on Lithium or Imipramine | Schizophrenia; SCAD | 122^¥^ | 36.4 | 24 | 66.8/35.2 | Li (1- other cause^); Placebo (1-suicide; 1-other cause^) | 55.0^¥^ | Yes** |
| Prien, Caffey’73 | Completed | USA | VA & non-VA Inpatient | BPAD, acute mania + stabilized on Lithium | None | 205 | 35.1 | 24 | 185/169 | Li (1- other cause^); Placebo (1-suicide; 1-other cause^) | 42.0 | Yes** |

BPD = Borderline Personality Disorder; BPAD = Bipolar Affective Disorder; ER = Emergency room; Li = Lithium; m = months; MDD = Major Depressive Disorder; MH = Mental Health; N = Number; PY = Person-years; Rx = Medication; SCAD = Schizoaffective Disorder; SUD = Substance use disorder; USA = United States of America;

VA = Department of Veterans Affairs

^§^ Suicidal behavior may include symptoms of suicidal ideation

^χ^ Calculation is based on the 54 patients for which there was information on baseline characteristics.

*Failed to respond to non-selective antidepressants or selective serotonin reuptake inhibitors

^¥^ Our review only included findings from the placebo and lithium arms (N=84).
^ The authors determined that the death was unrelated to the patients’ affective illness. No further details on the cause of death were provided in the publication.

**Lithium was supplied by Smith Kline & French Laboratories, Philadelphia (Prien studies) or SmithKline Beecham Pharmaceuticals (Bauer study).

**Supplementary Table 2 [Desired position – horizontal]** Risk of bias assessment of published studies.^¥^

|  | | | | | | | **Summary Conclusions** | |
| --- | --- | --- | --- | --- | --- | --- | --- | --- |
| **Author/**  **Year** | **Randomization Process** | **Effect of assignment** | **Effect of adherence** | **Missing data** | **Measurement of outcome** | **Selection of result** | **Key conclusions about risk of bias of included studies** | **Overall Risk** |
| Prien, Klett’73 | Some | Low | Some | Some | Low | Some | The authors provide limited information on the randomization process. The impact of loss to follow-up on the detection of mortality is unclear. Causes of death for non-suicide deaths are not described. The study is not posted on a trial registry, although the likelihood for reporting bias seems low. A high proportion of patients terminated early, but more dropouts occurred in the placebo arm. | Some |
| Prien, Caffey’73 | Some | Low | Some | Some | Low | Some | The authors provide limited information on the randomization process. The impact of loss to follow-up on the detection of mortality is unclear. The study is not posted on a trial registry, although the likelihood of reporting bias seems low. A high proportion of patients terminated early, but more dropouts occurred in the placebo arm. The authors report there were marked fluctuations in lithium levels in some patients, suggesting poor treatment adherence. | Some |
| Bauer’00 | Some | Low | Low | Low | Low | Some | There is limited information about the methods used for randomization. The study, however, reported that baseline characteristics were similar between arms. | Low |
| Lauterbach’08 | Some | Some | Low | Some | Low | Low | The study mentioned at baseline that there were a higher number of patients with personality disorder and history of multiple suicide attempts in the lithium arm. Yet, the placebo arm had a higher score on a suicide scale.  The study reported high attrition. The impact of loss to follow-up on the detection of mortality is unclear. The study was stopped early due to poor recruitment. Some patients may have been unmasked due to acute safety concerns requiring checking lithium levels. | Some |
| Khan’11 | Low | Low | Some | Low | Low | Low | Eleven patients had therapeutic levels and 29 patients had non-therapeutic levels. | Low |
| Girlanda’14 | Low | High | Low | Low | Low | Low | Study was at higher risk for bias because it used a treatment as usual control (rather than placebo). The study encountered recruitment challenges. The study was stopped early. | High |
| Katz’22 | Low | Some | Some | Low | Low | Low | The authors mentioned that there was a higher tendency for patients on lithium to correctly guess their assignment. Similarly, practitioners were fairly good at correctly guessing the assignment of patients on lithium. There were some concerns about poor lithium adherence and high attrition. The study encountered recruitment challenges. The trial was stopped early due to futility. The study had a robust strategy to detect mortality outcomes even in the setting of high attrition. | Some |

Low = Low risk of bias; High = High risk of bias; Some = some concern; RoB = Risk of bias

¥ Risk of bias assessed using the Cochrane Risk of Bias 2.0 Tool

**Supplementary Table 3: [Desired position – horizontal]** GRADE analysis of seven randomized controlled trials comparing lithium versus control for the prevention of death by suicide

| **Certainty assessment** | | | | | | | **Suicide incidence** | | **Effect Size** | | **Certainty** | **Importance** |
| --- | --- | --- | --- | --- | --- | --- | --- | --- | --- | --- | --- | --- |
| **№ of studies** | **Study design** | **Risk of bias** | **Inconsistency** | **Indirectness** | **Imprecision** | **Other considerations** | **Lithium** | **Control** | **Relative (95% CI)** | **Absolute (95% CI)** |  |  |
| 7 | randomised trials | not serious* | not serious | not serious | serious** | strong association | 2/568 (0.4%) | 8/570 (1.4%) | **OR 0.3** (0.09 to 1.02) | **10 fewer per 1,000** (from 13 fewer to 0 fewer) | ⨁⨁⨁◯ Moderate** | Important |

**CI:** confidence interval; **OR:** odds ratio
*We determined that the majority of the information came from studies at low or unclear risk of bias and that the potential limitations were unlikely to lower our confidence in the summary estimate
**We decided to downgrade the findings by one-level because the estimate was based on less than 10 studies (1,138 subjects) and the event rate was low.

**Supplementary Figure 1 [Desired position vertical]:** PRISMA Flow Diagram


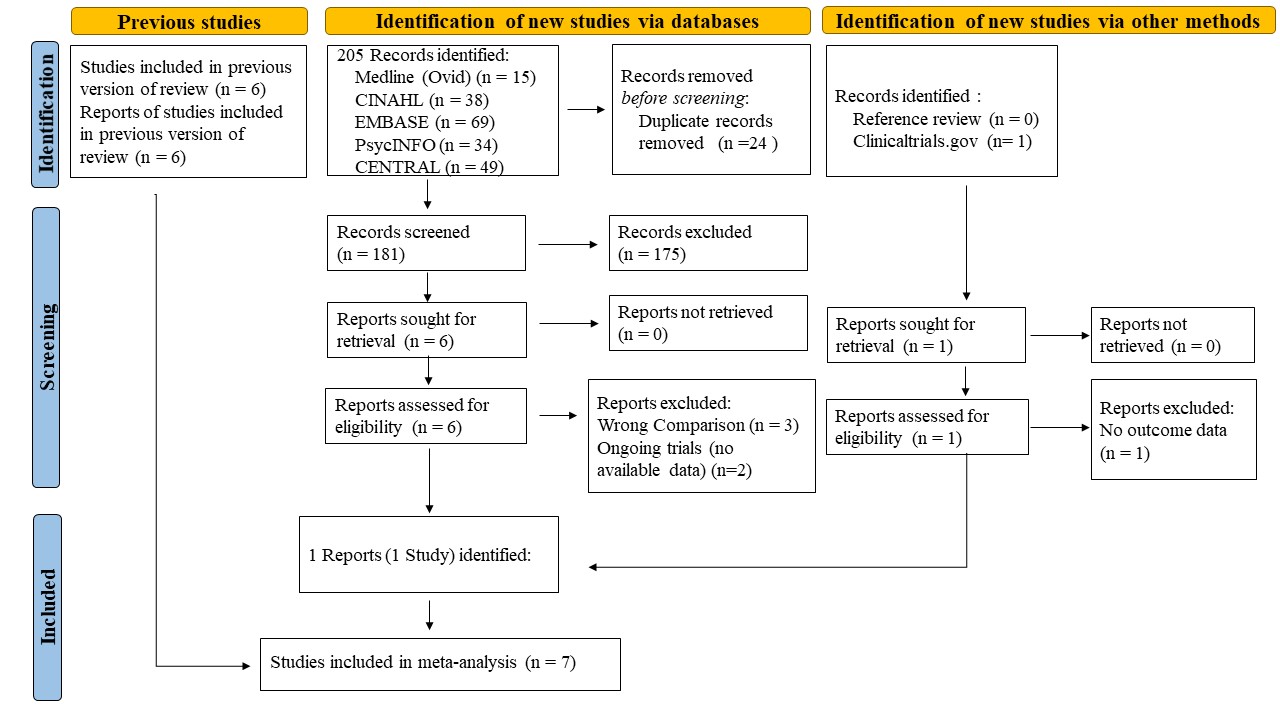


Legend:

CINAHL = The Cumulative Index to Nursing and Allied Health Literature

EMBASE = Excerpta Medica Database

CENTRAL = Cochrane Central Register of Controlled Trials

**Supplementary Figure 2:** Funnel plot of odds of death by suicide among six trials comparing lithium versus control.*


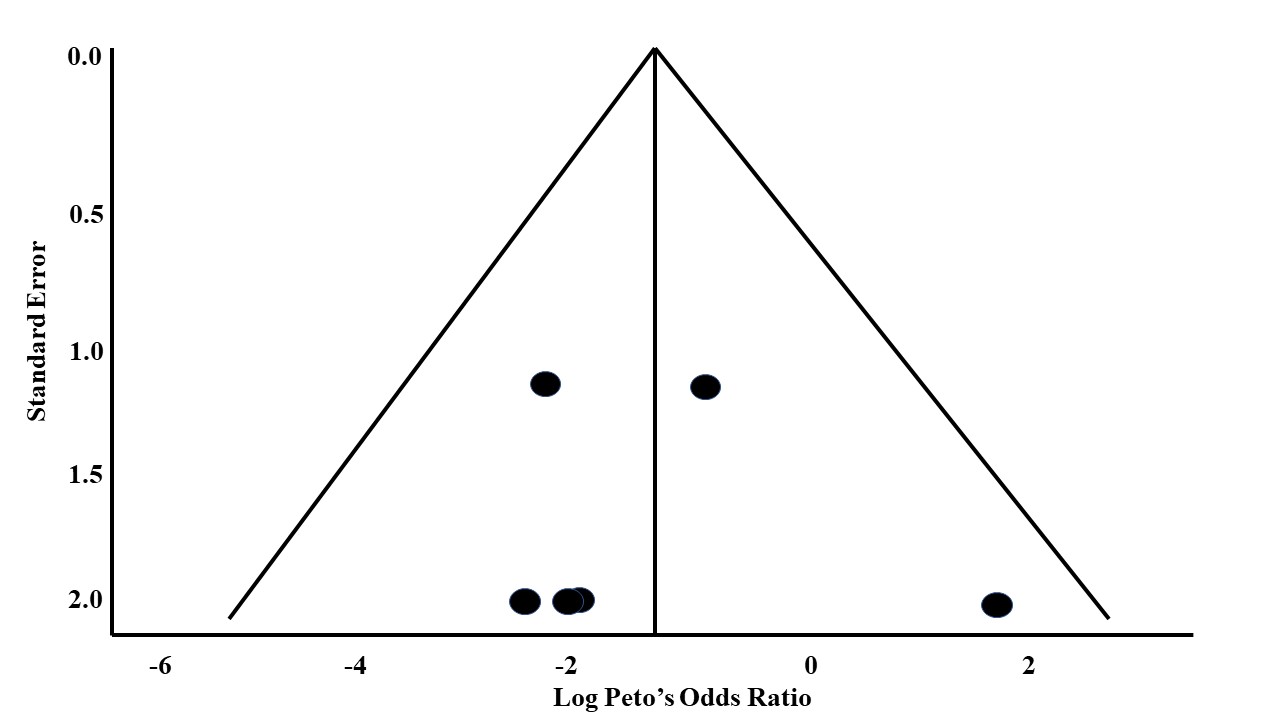


Legend:

*A seventh trial reported double zero events and therefore did not contribute to the outcome.
