# Supplementary material for: Lithium in the prevention of suicide in adults: systematic review and meta-analysis of clinical trials
Source: BJPsych Open. 2022 Nov 17;8(6):e199. doi: 10.1192/bjo.2022.605 (PMC9707499; doi:10.1192/bjo.2022.605)
Supplement: Supplementary file 1 [file bjosup.zip › S2056472422006056sup002.docx]

**Supplementary Materials**

**Methods**

We conducted our analysis according to standard methodology as described in the Cochrane Handbook.[^1^](#_ENREF_1) We reported our results according to the PRISMA guidelines.[^2^](#_ENREF_2) Because our systematic review serves as an update to a prior published meta-analysis by Riblet *et al.* (2017),[^3^](#_ENREF_3) we reported the results of our search using the PRISMA 2020 flow diagram for updated systematic reviews.^[2](#_ENREF_2)^

We submitted the protocol to Prospero on December 1, 2021. At the time of submission, we had not begun data abstraction. Because Prospero is currently experiencing a high demand, reviewers are instructed to continue with their review process. Our protocol was officially posted to the Prospero website on January 1, 2022 (CRD42022295822).

**Literature sources**

We searched with the intent of updating the results of a previous search completed by Riblet *et al.* (2017) in their meta-analysis of strategies to prevent suicide.[^3^](#_ENREF_3) Specifically, Riblet *et al.* (2017) searched Medline (via Ovid), Excerpta Medica Database (EMBASE), Cumulative Index to Nursing and Allied Health Literature (CINAHL), the Cochrane Central Register of Controlled Trials (CENTRAL), and PsycINFO from inception to 31 December 2015.[^3^](#_ENREF_3) This search yielded six randomized controlled trials (RCTs) of lithium.[^4-9^](#_ENREF_4) We updated these results by searching the same electronic databases from January 1, 2015 through November 30, 2021 to identify published articles (including Epub ahead of print) that met our study inclusion criteria. We deliberately allowed for one year of overlap in our search strategy to address any concerns that studies could have been indexed in 2015 after Riblet *et al.* (2017) completed their search.[^3^](#_ENREF_3)

To identify any studies that may have been missed in our primary search, we also reviewed the references of included studies and searched ClinicalTrials.gov.

**Search terms**

We used MeSH terms and key words to generate the following themes: suicide and lithium. We then used the Boolean term “AND” to find the intersection between these two themes. In addition, we applied a highly sensitive search strategy to identify RCTs in Medline (via Ovid).[^10^](#_ENREF_10) We used the following MeSH terms and key words to search Medline for potentially eligible studies.

*Search terms for the themes of suicide and lithium*

- Suicide.mp or Exp Suicide/ or Exp Suicide, Attempted/or Exp Suicide, Completed
- Suicidal ideation.mp or Exp Suicidal Ideation/
- Exp Antimanic Agents/pd [Pharmacology/]
- Exp Lithium
- Exp Lithium Carbonate
- Lithium.mp
- Lithium Citrate.mp

*Search terms for the highly sensitive search strategy to identify RCTs in Medline (via Ovid).^10^*

- (randomized controlled trial.pt OR controlled clinical trial.pt OR randomized.ab OR placebo.ab OR drug therapy.fs. OR randomly.ab OR trial.ab OR groups.ab) **AND** (not Exp animals/not humans.sh)

We modified the aforementioned terms as necessary to search the remaining electronic databases and ClinicalTrials.gov. As suggested in the literature, we applied a highly sensitive search strategy to identify RCTs in Excerpta Medica Database (EMBASE),[^10^](#_ENREF_10) Cumulative Index to Nursing and Allied Health Literature (CINAHL),[^11^](#_ENREF_11) and PsycINFO.[^12^](#_ENREF_12)

**Eligibility criteria**

We included studies that randomly assigned adult patients to lithium or a control condition (placebo, usual care, placebo or waitlist). We required that the study population were mostly 18 years or older. In addition, we required that studies reported on death by suicide as a primary or secondary outcome. Consistent with recommendations from the literature, we included studies even if there were no suicide events.[^13^](#_ENREF_13) We applied no language limits to our search.

We did not restrict our study population to a particular diagnostic condition or set of symptoms. We made this decision because there is evidence in the literature that lithium may have a role in suicide prevention in a range of populations. For example, population-level research has suggested an association between the trace element lithium in drinking water and lower suicide rates.[^14^](#_ENREF_14)

**Study identification and data abstraction**

One reviewer (NR) applied our study inclusion (and exclusion) criteria to the titles and abstracts of potentially eligible publications. Two reviewers (NR, BS) then independently assessed the full text of the remaining publications to make a final determination regarding study eligibility. Discrepancies were resolved by involving a third reviewer (BW) who independently evaluated the full texts to make a final determination about study inclusion.

For each study, demographics, methods, outcomes, and risk of bias were abstracted using a standardized data collection form. We used the Cochrane Risk of Bias Tool 2.0 to assess risk of bias.[^15^](#_ENREF_15)

The data abstraction was performed by two reviewers (NR, BS) independently and in duplicate. Discrepancies were resolved through consensus.

**Primary outcome and data analysis**

The outcome of interest was death by suicide. We did not examine other suicide-related outcomes such as suicidal ideation or non-fatal suicide attempts.

We evaluated the efficacy of lithium versus control for preventing death by suicide by calculating summary odds ratio (OR) with 95% confidence intervals (CI) and p-values. As recommended in the literature, we used the Peto Method to carry out our analysis because this method is more powerful in the case of a rare outcome.^16,17^ We did not apply a correction in the event that a trial reported double zero events (i.e. no event in either arm)[^6^](#_ENREF_6) because this is not recommended with the Peto Method.^16^

We considered that an OR that was smaller meant that the relative odds of suicide was reduced in the lithium versus control condition, and the opposite was true if the OR was larger than one. We defined an OR as statistically significant if the p-value was <0.05 and the 95% confidence interval did not cross one.

We assessed for heterogeneity using Cochrane’s Q and the I^2^ statistic. We defined substantial and significant heterogeneity using the typical threshold of p < 0.10 and an I^2^ > 50%.^16^

We conducted the analysis using STATA 17 (StataCorp).

**Confirmatory Analysis**

In order to address potential limitations of available meta-analytic methods to assess rare events, we carried out a confirmatory analysis.[^18^](#_ENREF_22) We used a Poisson regression model with random effects and calculated an incidence rate ratio (IRR) for suicides over person-year (PY).^19,20^ This method is useful because it can account for differences in exposure time across studies,^19,20^  addresses any potential heterogeneity between trials^20^ and better account for trials reporting zero events.^19,20^

Because PY may differ between study arms based on the number of assigned subjects and loss to follow-up, we calculated PY for each study assignment. In the event that the authors reported PY[^9^](#_ENREF_9) or mean total study follow-up^21^ for the lithium and control groups, we abstracted these data directly from the publication. Otherwise, we calculated PY by summing the total amount of follow-up time contributed by all the subjects assigned to the study arm.^22^ We assumed that complete follow-up data was available if the authors stated that there was no loss to follow-up[^6^](#_ENREF_6)^,^ [^7^](#_ENREF_7) or the authors stated that they had access to full data for our outcome of interest (i.e., suicide).[^8^](#_ENREF_8) In the case that a study reported loss to follow-up,[^4^](#_ENREF_4)^,^ [^5^](#_ENREF_5) we accounted for this variation in follow-up time in our calculations.

Prior to conducting our confirmatory analysis, we first performed a boundary likelihood-ratio test in order to assess our study data for overdispersion.^23^ We considered that there was over-dispersion if the alpha (the estimate of the dispersion parameter) was significantly larger than zero, defined as a *p*-value <0.05.^23^ We found that the alpha for our study data was equal to zero (χ^2^= 0.10, p-value 0.4), suggesting that it was appropriate to use a Poisson regression model.

We conducted the confirmatory analysis using STATA 17 (StataCorp).

**Reporting Bias**

In order to assess for publication bias, we generated a funnel plot for our primary outcome.^24^ We visually inspected the plot for asymmetry. We considered that a negative correlation between sample size and effect size (i.e., lack of small studies with negative findings) was suggestive of publication bias.^24^ As described earlier, we also searched ClinicalTrials.gov to identify any unpublished trials.

Because our meta-analysis included a total of seven studies, we did not perform a formal test for publication bias. The Cochrane Handbook cautions that tests for funnel plot asymmetry produce unreliable results in the case of fewer than 10 studies.^24^

**Quality Analysis**

We assessed the quality of the evidence and its impact on our overall results using the Grading of Recommendations Assessment, Development and Evaluation (GRADE) methodology.[^25^](#_ENREF_28) In our analysis, we considered risk of bias, inconsistency, indirectness, imprecision, the event rate, the effect size, certainty, importance and other factors including publication bias, magnitude of the effect size, dose-response gradient, and residual confounding.^25^

We conducted the GRADE analysis using GRADEpro software.^26^

**References**

1. Higgins JPT, Thomas J, Chandler J, Cumpston M, Li T, Page MJ, et al (editors). Cochrane Handbook for Systematic Reviews of Interventions [e-book]. Version 6.2 (updated February 2021). Cochrane; 2021 [cited 2021 Dec 12]. Available from www.training.cochrane.org/handbook.

2. Page MJ, McKenzie JE, Bossuyt PM, Boutron I, Hoffmann TC, Mulrow CD, et al. The PRISMA 2020 statement: an updated guideline for reporting systematic reviews. *BMJ* 2021;**372**: n71. doi:10.1136/bmj.n71.

3. Riblet NBV, Shiner B, Yinong Y-X, Watts BV, Young-Xu Y. Strategies to prevent death by suicide: meta-analysis of randomised controlled trials. *Br J Psychiatry* 2017;**210**(6):396-402. doi:10.1192/bjp.bp.116.187799

4. Prien RF, Caffey EM, Jr., Klett CJ. Prophylactic efficacy of lithium carbonate in manic-depressive illness. Report of the Veterans Administration and National Institute of Mental Health collaborative study group. *Arch Gen Psychiatry* 1973;**28**(3):337-41. doi:10.1001/archpsyc.1973.01750330035006

5. Prien RF, Klett CJ, Caffey EM, Jr. Lithium carbonate and imipramine in prevention of affective episodes. A comparison in recurrent affective illness. *Arch Gen Psychiatry* 1973;**29**(3):420-5. doi:10.1001/archpsyc.1973.04200030104017

6. Khan A, Khan SR, Hobus J, Faucett J, Mehra V, Giller EL, et al. Differential pattern of response in mood symptoms and suicide risk measures in severely ill depressed patients assigned to citalopram with placebo or citalopram combined with lithium: role of lithium levels. *J Psychiatr Res* 2011;**45**(11):1489-96. doi:10.1016/j.jpsychires.2011.06.016

7. Bauer M, Bschor T, Kunz D, Berghöfer A, Ströhle A, Müller-Oerlinghausen B. Double-blind, placebo-controlled trial of the use of lithium to augment antidepressant medication in continuation treatment of unipolar major depression. *Am J Psychiatry* 2000;**157**(9):1429-35. doi:10.1176/appi.ajp.157.9.1429

8. Girlanda F, Cipriani A, Agrimi E, Appino MG, Barichello A, Beneduce R, et al. Effectiveness of lithium in subjects with treatment-resistant depression and suicide risk: results and lessons of an underpowered randomised clinical trial. *BMC Res Notes* 2014;**7**:731. doi:10.1186/1756-0500-7-731

9. Lauterbach E, Felber W, Mueller-Oerlinghausen B, Ahrens B, Bronisch T, Meyer T, et al. Adjunctive lithium treatment in the prevention of suicidal behaviour in depressive disorders: a randomised placebo-controlled trial, 1-year trial. *Acta Psychiatrica Scandinavica* 2008;**118**:469-479.

10. Lefebvre C, Glanville J, Briscoe S, Littlewood A, Marshall C, Metzendorf M-I, et al. Technical supplement to Chapter 4: Searching for and selecting studies. In: Higgins JPT, Thomas J, Chandler J, Cumpston MS, Li T, Page MJ, Welch VA, editors. Cochrane Handbook for Systematic Reviews of Interventions Version 6.2 (updated February 2021) [e-book]. Cochrane; 2021 [cited 2021 Dec 15] Section 3.6.1; Section 3.6.2. Available from: www.training.cochrane.org/handbook.

11. Clowes, M. CINAHL for EBSCO. Search filters resources: Randomized controlled trials [Internet]. Healthcare Improvement Scotland SIGN; [cited 2021 Dec 15]. Available from: https://www.sign.ac.uk/what-we-do/methodology/search-filters/

12. Cochrane. PsycINFO: Identifying RCTs in PsycINFO: Search strategy, amended to ProQuest format [Internet]. Cochrane; 2021 [cited 2021 Dec 15]. Available from: https://work.cochrane.org/psycinfo.

13. Friedrich JO, Adhikari NK, Beyene J. Inclusion of zero total event trials in meta-analyses maintains analytic consistency and incorporates all available data. *BMC Med Res Methodol* 2007;**7**:5.

14. Lewitzka U, Severus E, Bauer R, Ritter P, Müller-Oerlinghausen B, Bauer M. The suicide prevention effect of lithium: more than 20 years of evidence-a narrative review. *International journal of bipolar disorders*. 2015;3(1):32-32. doi:10.1186/s40345-015-0032-2

15. Sterne JAC, Savović J, Page MJ, Elbers RG, Blencowe NS, Boutron I, et al. RoB 2: a revised tool for assessing risk of bias in randomised trials. *BMJ* 2019;**366**:l4898. doi:10.1136/bmj.l4898

16. Deeks JJ, Higgins JPT, Altman DG, (editors). Chapter 10: Analysing data and undertaking meta-analyses. In: Higgins JPT, Thomas J, Chandler J, Cumpston M, Li T, Page MJ, Welch VA (editors). Cochrane Handbook for Systematic Reviews of Interventions version 6.2 (updated February 2021) [e-book]. Cochrane; 2021 [cited 2021 Dec 15]: Section 10.4.2; Section 10.10. Available from www.training.cochrane.org/handbook.

17. Lane PW. Meta-analysis of incidence of rare events. *Stat Methods Med Res* 2013;**22**:117-132.

18. Shuster JJ, Walker MA. Low-event-rate meta-analyses of clinical trials: implementing good practices. *Stat Med* 2016; **35**(14): 2467-2478. doi: 10.1002/sim.6844

19. Cai T, Parast L, Ryan L. Meta-analysis for rare events. *Stat Med* 2010;**29**:2078-2089.

20. Spittal MJ, Pirkis J, Gurrin LC. Meta-analysis of incidence rate data in the presence of zero events. *BMC Med Res Methodol* 2015;**15**:1-16.

21. Katz IR, Rogers MP, Lew R, Thwin SS, Doros G, Ahearn E, et al. Lithium treatment in the prevention of repeat suicide-related outcomes in Veterans with major depression or bipolar disorder: A randomized clinical trial. *JAMA Psychiatry* 2022;**79**:24-32. doi:https://dx.doi.org/10.1001/jamapsychiatry.2021.3170

22. Alexander LK, Lopes B, Ricchetti-Masterson K, Yeatts KB. Calculating person-time [Internet]. Eric Notebook, Second Edition. UNC Gillings School of Global Public Health, UNC CH Department of Epidemiology; [cited 2021 Dec 15]. Available from: https://sph.unc.edu/wp-content/uploads/sites/112/2015/07/nciph_ERIC4.pdf.

23. STATA.com. nbreg - Negative binomial regression [Internet]. STATA; [cited 2021 Dec 15]. Available from https://www.stata.com/manuals/rnbreg.pdf.

24. Page MJ, Higgins JPT, Sterne JAC. Chapter 13: Assessing risk of bias due to missing results in a synthesis. In: Higgins JPT, Thomas J, Chandler J, Cumpston M, Li T, Page MJ, Welch VA (editors). Cochrane Handbook for Systematic Reviews of Interventions version 6.2 (updated February 2021) [e-book]. Cochrane; 2021 [cited 2021 Dec 15]: Section 13.3.5.2 – 13.3.6. Available from www.training.cochrane.org/handbook.

25. Schünemann H, Brożek J, Guyatt G, Oxman A, editors. GRADE handbook for grading quality of evidence and strength of recommendations [Internet]. Updated October 2013. The GRADE Working Group; 2013 [cited 2021 Dec 14]. Available from guidelinedevelopment.org/handbook.

26. GRADEpro GDT: GRADEpro Guideline Development Tool [Software]. McMaster University and Evidence Prime, 2021 [cited 2021 Dec 14]. Available from gradepro.org.
